# Claims-Based Definition of Death in Japanese Claims Database: Validity and Implications

**DOI:** 10.1371/journal.pone.0066116

**Published:** 2013-05-31

**Authors:** Nobuhiro Ooba, Soko Setoguchi, Takashi Ando, Tsugumichi Sato, Takuhiro Yamaguchi, Mayumi Mochizuki, Kiyoshi Kubota

**Affiliations:** 1 Department of Pharmacoepidemiology, Graduate School of Medicine, University of Tokyo, Tokyo, Japan; 2 Duke Clinical Research Institute, Durham, North Carolina, United States of America; 3 Division of Evaluation and Analysis of Drug Information, Keio University Faculty of Pharmacy, Tokyo, Japan; 4 Drug Safety Research Unit Japan, Tokyo, Japan; 5 Department of Public Health and Forensic Medicine, Tohoku University Graduate School of Medicine, Miyagi, Japan; Old Dominion University, United States of America

## Abstract

**Background:**

For the pending National Claims Database in Japan, researchers will not have access to death information in the enrollment files. We developed and evaluated a claims-based definition of death.

**Methodology/Principal Findings:**

We used healthcare claims and enrollment data between January 2005 and August 2009 for 195,193 beneficiaries aged 20 to 74 in 3 private health insurance unions. We developed claims-based definitions of death using discharge or disease status and Charlson comorbidity index (CCI). We calculated sensitivity, specificity and positive predictive values (PPVs) using the enrollment data as a gold standard in the overall population and subgroups divided by demographic and other factors. We also assessed bias and precision in two example studies where an outcome was death. The definition based on the combination of discharge/disease status and CCI provided moderate sensitivity (around 60%) and high specificity (99.99%) and high PPVs (94.8%). In most subgroups, sensitivity of the preferred definition was also around 60% but varied from 28 to 91%. In an example study comparing death rates between two anticancer drug classes, the claims-based definition provided valid and precise hazard ratios (HRs). In another example study comparing two classes of anti-depressants, the HR with the claims-based definition was biased and had lower precision than that with the gold standard definition.

**Conclusions/Significance:**

The claims-based definitions of death developed in this study had high specificity and PPVs while sensitivity was around 60%. The definitions will be useful in future studies when used with attention to the possible fluctuation of sensitivity in some subpopulations.

## Introduction

Large claims databases have been widely used in pharmacoepidemiology studies in US and Europe for the past couple of decades [1] and more recently in Asian countries such as Taiwan and Korea [2], [3]. In Japan, the National Data Base (NDB) of healthcare claims covering the entire population was recently developed and has accumulated data since 2009. The Japanese Ministry of Health, Labour and Welfare (MHLW) made a part of the NDB available to selected researchers for pilot research projects in April 2011 [4]. However, researchers will not have access to its enrollment files that include vital status and date of death. Furthermore, the database does not contain unique identifiers and the MHLW currently prohibits attempts for linkage to vital statistics, medical records, and other data sources. The lack of death information may pose significant challenges in using the NDB to study safety and effectiveness of medications and medical devices.

In the current study, we developed claims-based definitions of death and assessed their validity using death information from enrolment files in a commercially available claims database in Japan. In addition, the practical implications of using the claims-based definitions were evaluated in two example studies.

## Methods

### Data sources and study patients

Healthcare utilization data for 195,193 beneficiaries aged between 20 and 74 years from three private health insurance unions (Unions 1 to 3) were made available for this study through a database vendor, Japan Medical Data Center Co., Ltd [5]. We observed at least one claim in 167,710 beneficiaries during the study period (1 January 2005 to 31 August 2009). Diagnostic information was coded using the 10th revision of the international classification of diseases (ICD-10). The data also provided inpatient and outpatient drug dispensing, which was coded by National Health Insurance Drug Price Standard Code, a drug coding system used in Japanese health insurance system. The data also provided discharge status in inpatient claims and the potential values include ‘death’, ‘cure’, ‘termination’ and ‘others’. Except for ‘death’, the value indicates the status of provisions of health care rather than the disease outcome: ‘cure’ means that no further health care is needed because of complete cure or improvement, ‘termination’ means that no health care will be provided at least for the time being (e.g., transfer to another hospital or discharge due to patient’s refusal of care) and ‘others’ indicates continued therapy (in the claim issued monthly even if the patient is hospitalized for months). Similar information to inpatient discharge status is also available in outpatient claims (disease status classified into ‘death’, ‘cure’, ‘termination’ and ‘others’).

### Claims-based definition of death

We identified all in- and out-patient claims with discharge or disease status indicated as ‘death’ and defined them as the index claim. Contrary to expectations that the index claim should be the last claim for the patient, we occasionally found claims with the discharge/disease status not specified as ‘death’ (defined as ‘zombie’ claims) one or more months after the index claim was issued for the patient. To take this paradoxical situation into consideration, we developed 3 variations of claims-based definition excluding none, all and some of the patients with ‘zombie’ claims. (Definition 1.1, 1.2, and 1.3 in Table 1). Another set of definitions of death used information from inpatient claims only using the ICD-10 adaptation of the Charlson comorbidity index (CCI) [6]: those whose CCI calculated from the last inpatient claim was ≥6 (Definition 2.1), those who met Definition 2.1 and their index claim was followed by the blank period (period without any claim) for at least 6 months before the end of the observation period (Definition 2.2), and those who met Definition 2.2 and had CCI ≥6 in one or more claims issued within 12 months preceding the index claim (Definition 2.3). Finally, we assessed the validity of definitions combining Definition 1.3 and 2: those who met Definition 1.3 or 2.1 (Definition 3.1), Definition 1.3 or 2.2 (Definition 3.2) and Definition 1.3 or 2.3 (Definition 3.3). (Table 1)

**Table 1:** Claims-based definitions of death.

Definition	Description	Contents
Definition 1.1	‘Dead’ on claim	Those with claims indicating ‘death’ as discharge/disease status
Definition 1.2	‘Dead’ on claim excluding ‘zombie’	Definition 1.1 and no ‘zombie’ claims issued after the date of the claim indicating ‘death’
Definition 1.3	‘Dead’ on claim excluding long-term ‘zombie’	Definition 1.1 and no ‘zombie’ claims issued in > 2 months after the date of the claim indicating ‘death’
Definition 2.1	Admitted for serious condition	CCI ≥6 for the last inpatient claim
Definition 2.2	Admitted for serious condition followed by blank period (no health care service≥6 m)	Definition 2.1 + period with no claim to end of study ≥6 m
Definition 2.3	Admitted for long serious condition followed by blank period (≥6 m)	Definition 2.2 + CCI ≥6 in 1 or more claims in preceding 12 m
Definition 3.1	Death on claim or serious condition	Definition 1.3 or 2.1
Definition 3.2	Death on claim or serious condition + blank period	Definition 1.3 or 2.2
Definition 3.3	Death on claim or prolonged serious condition + blank period	Definition 1.3 or 2.3

Abbreviation: CCI, charlson comorbidity index.

### Gold standard death information from enrollment files

For the 195,193 study patients, we also obtained the enrollment data through the same database vendor and used them as the gold standard information for death. The enrollment data contained age, sex, type of beneficiary (employee or family member), date (as year and month abbreviated as year/month) of enrollment, the year/month and reason of disenrollment, and date of death. Using the enrollment data, the end of the observation period was defined as the date of disenrollment or 31 August 2009 whichever came first.

### Sensitivity, specificity and positive predictive values (PPVs)

We calculated sensitivity, specificity and PPVs of the claims-based definition of death in the entire population and subgroups defined by age and sex, type of beneficiary (employee/family member), history of admission due to any reason in one year preceding the end of observation, origin of the index claim (inpatient vs. outpatient), comorbidity (cancer, diabetes mellitus, hypertension, depression and hyperlipidemia) and use of drugs (anticancer drugs, antidiabetics, antihypertensives, selective serotonin reuptake inhibitors (SSRIs), other oral antidepressants, statins, and non-steroidal anti-inflammatory drugs (NSAIDs) prescribed/dispensed at least once during the study period (vs. not prescribed/dispensed at all).

### Implications of claims-based definitions in example studies

To understand the potential impact of misclassification and loss of precision associated with the use of the claims-based definition of death in the studies where death is an outcome and mortality is compared between users of medications, we have conducted two example studies. In one study, we compared mortality between antipyrimidines (fluorouracil, tegafur and others) and platinum compounds (cisplatin, carboplatin and others) in patients with the diagnosis code of digestive organ cancer who newly (after 6-months of non-use) started the drug. In another study, mortality was compared between a group of patients who newly started selective serotonin reuptake inhibitors (SSRIs) and another group who newly started other oral antidepressants. In both studies, we compared the hazard ratio (HR) estimated by using the claims-based definition of death to those using death information from the enrollment data (gold standard).

The HR and its 95% confidence intervals (CIs) adjusted for age and sex were calculated by the Cox regression model. All analyses were performed using version 9.2 of the SAS system for Windows (copyright, SAS Institute Inc., Cary NC, USA). This study was approved by the ethics committee of the Tokyo University Graduate School and Faculty of Medicine (No. 3927). We used anonymized data with serial study IDs created by the data vendor.

## Results

Among the 195,193 beneficiaries included in the study, 60% were male with average age of 39.2 years old with 11% being older than 60 years old (Table 2). Approximately 60% were employees and 40% were family members. Comparing characteristics of the patients among 3 health insurance unions, the distribution of gender was similar. The age distribution was slightly different and the standardized difference [7] of the average age between any 2 of 3 unions was 0.13 to 0.35. Based on the gold standard vital status information from the enrollment data, 680 died during average follow-up of 2.0 years.

**Table 2:** Characteristics of beneficiaries in three health insurance unions.

Characteristics	Totaln = 195,193		Union 1n = 28,324		Union 2n = 99,681		Union 3n = 67,188	
	N	%	N	%	N	%	N	%
Male	116,932	59.9	17,509	61.8	61,394	61.6	38,029	56.6
Age (years)								
20−39	112,703	57.7	15,212	53.7	64,166	64.4	33,325	49.6
40−59	60,864	31.2	9,926	35.0	27,629	27.7	23,309	34.7
60−74	21,626	11.1	3,186	11.2	7,886	7.9	10,554	15.7
Average	39.2		40.1		37.2		41.9	
Type of beneficiary								
Employee	128,316	65.7	17,137	60.5	68,103	68.3	43,076	64.1
Family member	66,877	34.3	11,187	39.5	31,578	31.7	24,112	35.9
Observation (months)	36.1		43.4		40.0		27.3	
Claim issued	167,710	85.9	25,440	89.8	88,626	88.9	53,644	79.8

We identified 413 patients ‘dead’ by Definition 1.1 (based on the discharge or disease status in in- and out-patients claims). Of those, 13 had ‘zombie’ claims during 1 to 36 months following the index claim indicating death. Of those, 4 patients (31%) did not die according to the gold standard while 9 patients (69%) were dead by both Definition 1.1 and the gold standard information. For 14 patients ‘dead’ by Definition 1.1 with no ‘zombie’ claim, the enrollment data indicated disenrollment in the year/month when the index claim was issued but the reason for disenrollment was not specified as death and they were considered to be false-positive cases in Table 3. Sensitivity was 57 to 58%, specificity was 99.99% and PPVs were 96 to 97% for these definitions using information of discharge or disease status only (Definitions 1.1 to 1.3, Table 4).

**Table 3:** Deaths identified by the gold standard definition and Definition 1.1*.

Death in enrollment data (Gold standard information) ‡	Death by Definition 1.1*					Total
	Yes Status of ‘zombie’ claims†				No	
	No	short-term¶	long-term§	subtotal		
Yes	386	9	0	395	285	680
No	14	0	4	18	194,495	194,513
Total	400	9	4	413	194,780	195,193

* Definitions 1.1 given in Table 1.

† ‘Zombie’ claims are the claims without ‘death’ issued after the index claim with ‘death’ given as the discharge/disease status.

‡ Information on death in enrollment file provided by insurers was considered to be the gold standard information.

¶ ‘Zombie’ claims issued up to 1 or 2 months after the index claim.

§ ‘Zombie’ claims issued up to 3 or more months after the index claim.

**Table 4:** Sensitivity, specificity and PPVs of claims-based definition of death.

Definition *	Description	N of patients meeting a definition (True positive)	Sensitivity (95%CI)	Specificity (95%CI)	PPV (95%CI)
Gold Standard		680	−	−	−
Definition 1.1	‘Dead’ on claim	413 (395)	58.1 (54.3−61.8)	99.99 (99.99−99.99)	95.6 (93.2−97.4)
Definition 1.2	‘Dead’ on claim excluding 'zombie'	400 (386)	56.8 (53.0−60.5)	99.99 (99.99−100)	96.5 (94.2−98.1)
Definition 1.3	‘Dead’ on claim excluding long-term 'zombie'	409 (395)	58.1 (54.3−61.8)	99.99 (99.99−100)	96.6 (94.3−98.1)
Definition 2.1	Admitted for serious condition	290 (215)	31.6 (28.1−35.3)	99.96 (99.95−99.97)	74.1 (68.7−79.1)
Definition 2.2	Admitted for serious condition + blank period	218 (194)	28.5 (25.2−32.1)	99.98 (99.98−99.99)	89.0 (84.1−92.8)
Definition 2.3	Admitted for long serious condition + blank period	167 (155)	22.8 (19.6−26.0)	99.99 (99.99−100)	92.8 (87.8−96.2)
Definition 3.1	Definition 1.3 or 2.1	506 (424)	62.4 (58.6−66.0)	99.96 (99.95−99.97)	83.8 (80.3−86.9)
Definition 3.2	Definition 1.3 or 2.2	453 (420)	61.8 (58.0−65.4)	99.98 (99.98−99.99)	92.7 (89.9−94.9)
Definition 3.3	Definition 1.3 or 2.3	442 (419)	61.6 (57.8−65.3)	99.99 (99.98−99.99)	94.8 (92.3−96.7)

Abbreviation: PPV, positive predictive value; 95% CI, 95% confidence interval.

* Definitions are given in Table 1.

Of 285 subjects whose death was noted in the enrollment data but not in the claims, 66 (23%) were young (20−39 years old) while 43 (11%) of 395 subjects whose death was given in claims were young and the standardized difference was 0.33. The proportion of old subjects (60−74 years old) was essentially the same and 36% in these two groups (102/285 versus 142/395, standardized difference was 0.003). In 66 young subjects whose death was not in the claims data, 2 (3%) had the diagnosis of cancer, while in 43 young subjects whose death was in the claims data, 18 (42%) had cancer. Otherwise, we could not find any difference of the distribution of demographic and other factors which may be contributory to the low sensitivity when deaths in the claims and those not in the claims were compared.

The CCI calculated from the last inpatient claim was 6 or more in the last inpatient claim in 290 inpatients. In 218 of the 290 patients, the last inpatient claim was followed by the blank period of 6 or more months where any kind of claim was not issued before the end of the study period (31 August 2009). The enrollment data confirmed death for 194 of these 218 patients. While the definitions using only CCIs had relatively lower sensitivity of 23 to 32% (Definitions 2.1 to 2.3, Table 4), the definitions using the combination of discharge or disease status and CCI from inpatient claims (Definitions 3.1 to 3.3) had the highest sensitivity (around 62%) without substantial loss of PPV (84 to 95%) and specificity (99.96 to 99.99%) as compared to those for Definitions 1.1 to 1.3 (Table 4).

Table 5 shows sensitivity, specificity and PPVs for Definition 3.3 in subgroups categorized by demographic and other factors. Sensitivity was around 60% but varied from 27.5 to 90.7%. For example, sensitivity was low (<40%) in young males, those whose last claim was outpatient and those who used SSRIs with or without other antidepressants, whereas it was high (>80%) in those hospitalized in the preceding year, those with diagnosis of cancer and those dispensed drugs for cancer, diabetes, hypertension and depression (excluding SSRI). Specificity and PPVs were high (>98% and >88%, respectively) in all of the subgroups.

**Table 5:** Sensitivity, specificity and PPVs for claims-based Definition 3.3*.

Characteristic	N of deaths in the insurer’s enrollment record†	Sensitivity (95%CI)	Specificity (95%CI)	PPV (95%CI)
Total	680	61.6 (57.8−65.3)	99.99 (99.98−99.99)	94.80 (92.29−96.67)
Males	445	59.1 (54.4−63.7)	99.98 (99.97−99.99)	93.26 (89.68−95.90)
Age (years)				
20−39	82	30.5 (20.8−41.6)	99.99 (99.99−100)	96.15 (80.36−99.90)
40−59	210	67.6 (60.8−73.9)	99.98 (99.96−99.99)	95.95 (91.39−98.50)
60−74	153	62.8 (54.6−70.4)	99.91 (99.83−99.95)	88.89 (81.40−94.13)
Females	235	66.4 (60.0−72.4)	99.99 (99.99−100)	97.50 (93.72−99.31)
Age (years)				
20−39	27	66.7 (46.0−83.5)	99.99 (99.99−100)	94.74 (73.97−99.87)
40−59	117	72.7 (63.6−80.5)	100.00 (99.99−100)‡	100.00 (95.75−100)‡
60−74	91	58.2 (47.4−68.5)	99.97 (99.90−99.99)	94.64 (85.13−98.88)
Type of beneficiary				
Employee	403	60.1 (55.1−64.9)	99.99 (99.98−99.99)	93.44 (89.70−96.13)
Family member	277	63.9 (57.9−69.6)	99.99 (99.99−100)	96.72 (93.00−98.79)
Hospitalized in preceding year	399	84.7 (80.8−88.1)	100.00 (99.95−100)‡	100.00 (98.91−100)‡
continued				
Last claims				
Outpatient	193	27.5 (21.3−34.3)	99.99 (99.99−100)	94.64 (85.13−98.88)
Inpatient	418	87.6 (84.0−90.6)	99.25 (98.84−99.54)	94.82 (92.11−96.81)
Diagnosis				
Cancer	344	85.2 (81.0−88.8)	99.93 (99.88−99.96)	94.52 (91.36−96.77)
Diabetes	216	78.7 (72.6−84.0)	99.95 (99.91−99.98)	94.44 (90.02−97.30)
Hypertension	222	77.9 (71.9−83.2)	99.97 (99.93−99.99)	96.65 (92.85−98.76)
Depression	90	58.9 (48.0−69.2)	99.99 (99.94−100)	98.15 (90.11−99.95)
Hyperlipidemia	127	65.4 (56.4−73.6)	99.97 (99.94−99.99)	92.22 (84.63−96.82)
Drugs				
Anticancers	194	90.7 (85.7−94.4)	98.67 (97.63−99.33)	94.12 (89.72−97.03)
Antidiabetics	101	81.2 (72.2−88.3)	99.91 (99.77−99.98)	95.35 (88.52−98.72)
Antihypertensives	204	82.4 (76.4−87.3)	99.97 (99.92−99.99)	97.11 (93.38−99.06)
SSRIs ¶	38	36.8 (21.8−54.0)	100.00 (99.94−100)‡	100.00 (76.84−100)‡
Oral antidepressants §	36	83.3 (67.2−93.6)	100.00 (99.63−100)‡	100.00 (88.43−100)‡
Statins	47	70.2 (55.1−82.7)	99.98 (99.93−100)	94.29 (80.84−99.30)
NSAIDs	422	75.1 (70.7−79.2)	99.99 (99.98−99.99)	94.91 (91.98−97.01)

Abbreviation: PPV, positive predictive value; 95%CI, 95% confidence interval; SSRI, selective serotonin reuptake inhibitor; NSAID, non-steroidal anti-inflammatory drug.

* Definition 3.3 in Table 1.

† The gold standard information.

‡ Definition yielded no false positive cases.

¶ SSRIs with or without other oral antidepressants.

§ Oral antidepressants except for SSRIs.

Table 6 shows the incidence rates and HRs and their 95% CIs in the two example studies comparing mortality in drug users. In Study 1 where mortality was compared between two anticancer drug classes, the point estimates of HR (0.83 and 0.71) and precisions (defined as the inverse of the variance of logarithm) of HR (7.3 and 7.7) were of similar magnitude between two definitions of death (claims-based definition (Definition 3.3 in Table 1) and gold standard definition. In Study 2 where mortality was compared between patients with SSRI and those with other antidepressants, the HR with the claims-based definition (0.10) was lower than that with the gold standard definition (0.27). The precision of HR with the claims-based definition (5.4) was also lower than that by the gold standard (12.5). It was noteworthy that in 268 of 878 patients with non-SSRI antidepressants and 799 of 3362 patients with SSRIs had diagnosis code of cancer and the proportion of deaths occurring in cancer patients with non-SSRI antidepressants (28/268, 10.4%) was 10 times greater than that in those with SSRIs (8/799, 1.0%).

**Table 6:** Hazard ratio for death in new user of anticancer drugs or antidepressants drugs.

	Patients with diagnosis of cancer of digestive organs		Patients with antidepressants	
	Anticancer drugs		SSRIs	Other drugs
	Antipyrimidines	Platinums		
N of patients	146	27	3,362	878
Death				
Claims-based definition *	43	9	7	27
Gold standard †	42	10	23	30
Death rate given in per 100 person-years (95% CI)				
Claims-based definition *	19.7 (14.6−26.6)	30.5 (15.9−26.6)	0.12 (0.05−0.24)	1.86 (1.25−2.67)
Gold standard †	19.3 (14.3−26.1)	34.0 (18.3−63.2)	0.40 (0.26−0.59)	2.07 (1.42−2.91)
Hazard ratio (95% CI) ‡				
Claims-based definition *	0.83 (0.40−1.72)	1.0 (Reference)	0.10 (0.04−0.22)	1.0 (Reference)
Gold standard †	0.71 (0.35−1.43)	1.0 (Reference)	0.27 (0.16−0.48)	1.0 (Reference)

Abbreviation: SSRI, selective serotonin reuptake inhibitor; 95% CI, 95% confidence interval.

* Definition 3.3 in Table 1 is used.

† Information of death in the insurer’s enrollment data is used.

‡ Adjusted for age and sex by Cox regression model.

## Discussion

Japan has recently created the national claims database covering the entire population. However, the enrollment file that contains information on death and personal identifiers is not made for research use. Therefore, using a commercially available claims database covering working population, we developed claims-based definitions of death and assessed sensitivity, specificity and PPV compared to the gold standard death information obtained from the enrollment data. Our claims-based definitions had very high specificity (>98%), a high PPV (>88%) but moderate sensitivity (∼60%) that varied among subgroups defined by comorbidity, drug use and others (28 to 91%). Of two example studies, claims-based definition of death gave HR and its 95% CI near to those by the gold standard definition of death in patients with anticancer drugs in Study 1 but claims-based definition of death gave biased and less precise estimates of HR in Study 2 where different classes of antidepressants were compared.

The criterion CCIs ≥ 6 used in Definitions 2.1 to 2.3 in this study was shown to predict death rate of 20 to 25% in hospitalized patients in a study conducted in Australia [6]. Another study in Australia showed that 30 to 180-day death proxy had sensitivity and specificity of 90% or more in adult cancer patients where the proxy indicated death if the difference between the last dispensing record and the end of the observational period exceeded the proxy cutoff [8]. The current study revealed that the combination of the index for ‘dead’ on claims (Definition 1.3) and that for CCIs and 6-month cutoff in the inpatient claim (Definition 2.3) may be used as a composite definition of death (Definition 3.3) to obtain the better sensitivity in researches using Japanese claims data.

We observed that one or more ‘zombie’ claims were issued after the index claim. As shown in Table 3, there seem to be at least two mechanisms to yield ‘zombie’ claims. Short-term ‘zombie’ claims issued 1 or 2 months after the index claim were presumably due to the delay of reimbursement processes for some kinds of health care services because they were issued for patients whose death was confirmed by the enrollment data file. On the other hand, long-term ‘zombie’ claims issued more than 2 months after the index claim probably indicated that the patient was in fact alive and the index claim was issued by some mistake such as miscoding discharge/disease status when the claim was issued in the medical institutions.

Of our two example studies, Study 1 compared mortality between two classes of anticancer drugs in cancer patients and those subgroups in general had high sensitivity as in Table 5. On the other hand, Study 2 compared different classes of antidepressants and one third of patients with non-SSRI antidepressants were likely to be those with advanced cancer who were prescribed the antidepressant to control chronic cancer pain [9]. Therefore, two patient groups compared in Study 2 might represent different subgroups in terms of sensitivity of claims-based definition of death. Those with non-SSRI antidepressants included those with advanced cancer in which claims-based definition of death had high sensitivity. On the other hand, in those with SSRIs, claims-based definition had low sensitivity as in young males (Table 5). One possible explanation for the reason why claims-based definition of death gave low sensitivity in patients with SSRIs and young males would be that deaths in those subgroups occurred outside hospital without using health care services. Indeed, 64% of all deaths in young males (20-39 years old) in the national vital statistics of 2008 [10] were due to traffic and other accidents or suicide. It is possible that suicide is one of the leading causes of death in young patients and those with SSRIs in our study as well. The use of claims-based definition of death may not be appropriate in the studies on deaths in those subpopulations where sensitivity of the definition is low. In particular, one may have to be careful not to compare mortality using the claims-based definition in two subpopulations with different level of sensitivity as in Study 2.

Our results should be interpreted in the light of several limitations. First, our study population did not include subjects aged 19 years or younger and 75 years or older. Also, because the data were for the beneficiaries of large health insurance unions, the study population was representative of younger and working population and their family members covered by private health insurance unions but not representative of older subjects or unemployed younger population. However, we have the universal health care system, which provide universal access to care with relatively low and similar out-of-pocket payments [11]. Nevertheless, further studies using the data of different types of health insurance unions and the data covering the whole range of age and sex are warranted. Second, sensitivity might be underestimated. Fourteen cases with the index claim were considered to be false positive because the enrollment data did not specify the cause of disenrollment as death. However, the year/month of these index claims was the same as that of disenrollment. The contents of enrollment data are maintained independently of the claims data by each insurer and it is possible that the reason of enrollment is amended as death for some or all of these 14 cases in the future update of the enrollment data.

In conclusion, we developed claims-based definitions of death, which were shown to have moderate sensitivity (around 60%) and high specificity (99.99%) and PPVs (94.8%). Among subgroups categorized by demographic factors, comorbidity status and treatment, the specificity and PPV remained very high but sensitivity varied from 28 to 91%. Our example studies indicated that the claims-based definition of death when used as an outcome could yield minimally biased estimates when conducted in the population where the definition gave high sensitivity and misclassification is minimal and non-differential. However, when misclassification is expected to be differential, e.g., an exposure may increase the risk of a cause of death that is more or less likely to be missed by the definition, it could yield biased results. Further studies are needed to assess the validity and implication of our definition in subjects not studied in the current study.
